# Activation of Nrf2 by Esculetin Mitigates Inflammatory Responses through Suppression of NF-κB Signaling Cascade in RAW 264.7 Cells

**DOI:** 10.3390/molecules27165143

**Published:** 2022-08-12

**Authors:** Thanasekaran Jayakumar, Chun-Jen Huang, Ting-Lin Yen, Chih-Wei Hsia, Joen-Rong Sheu, Periyakali Saravana Bhavan, Wei-Chieh Huang, Cheng-Ying Hsieh, Chih-Hsuan Hsia

**Affiliations:** 1 Department of Ecology and Environmental Sciences, Pondicherry University, Puducherry 605014, India; 2 Graduate Institute of Medical Sciences, College of Medicine, Taipei Medical University, Taipei 110, Taiwan; 3 Department of Anesthesiology and Integrative Research Center for Critical Care, Wan Fang Hospital, Taipei Medical University, Taipei 110, Taiwan; 4 Department of Medical Research, Cathay General Hospital, Taipei 106, Taiwan; 5 Department of Pharmacology, School of Medicine, College of Medicine, Taipei Medical University, Taipei 110, Taiwan; 6 Department of Zoology, Bharathiar University, Coimbatore 641046, India; 7 Translational Medicine Center, Shin Kong Wu Ho-Su Memorial Hospital, Taipei 111, Taiwan

**Keywords:** esculetin, NO/iNOS, MAPK/NF-κB, Nrf2, DPPH, anti-inflammation

## Abstract

Inflammation is a major root of several diseases such as allergy, cancer, Alzheimer’s, and several others, and the present state of existing drugs provoked researchers to search for new treatment strategies. Plants are regarded to be unique sources of active compounds holding pharmacological properties, and they offer novel designs in the development of therapeutic agents. Therefore, this study aimed to explore the anti-inflammatory mechanism of esculetin in lipoteichoic acid (LTA)-induced macrophage cells (RAW 264.7). The relative expression of inducible nitric oxide synthase (iNOS), nitric oxide (NO) production and COX-2 expression were intensified in LTA-induced RAW cells. The phosphorylation status of mitogen-activated protein kinases (extracellular signal-regulated kinase (ERK)1/2, p38 MAPK, and c-Jun N-terminal kinase (JNK)) and nuclear factor kappa B (NF-κB) p65 were detected by using Western blot assay. The nuclear translocation of p65 was assessed by confocal microscopic image analysis. Esculetin significantly and concentration-dependently inhibited LTA-induced NO production and iNOS expression, but not COX-2 expression, in RAW cells. Esculetin was not effective in LTA-induced MAPK molecules (ERK, p38 and JNK). However, esculetin recovered LTA-induced IκBα degradation and NF-κB p65 phosphorylation. Moreover, esculetin at a higher concentration of 20 µM evidently inhibited the nuclear translocation of NF-κB p65. At the same high concentration, esculetin augmented Nrf2 expression and decreased DPPH radical generation in RAW 264.7 cells. This study exhibits the value of esculetin for the treatment of LTA-induced inflammation by targeting NF-κB signaling pathways via its antioxidant properties.

## 1. Introduction

Inflammation and oxidative stress are known to play vital roles in the pathogenesis of several diseases. Numerous inflammatory mediators, such as nitric oxide (NO), iNOS, tumor necrosis factor-α (TNF-α), and interleukin 16 (IL-16), have been implicated in LPS-induced inflammation. Inducible nitric oxide synthase (iNOS) controls NO, a gaseous free radical. Elevated NO production can cause DNA damage and mutagenesis and result in cancer progression [1]. A study stated that IL-6, a main pro-inflammatory cytokine, induces the expression of iNOS, which ultimately augments NO production [2]. Furthermore, inflammation-induced excess generation of reactive oxygen species (ROS) can damage vital cellular components and induce oxidative stress [3]. Thus, finding compounds that can suppress the anomalous production of inflammatory mediators could hypothetically relieve inflammation-related chronic diseases.

Gram-positive bacterial infection, such as by pathogenic *Staphylococcus aureus* (*S. aureus*) and *Streptococcus pneumoniae* (*S. pneumoniae*), leads to acute lung injury, which is a world-leading cause of mortality [4]. Lipoteichoic acid (LTA) is a Gram-positive bacterial cell wall component and is considered a potential inducer of the immune response [5]. Because of its properties that might cause serious inflammatory disorders resulting from excessive stimulation of the host immune system, LTA is regarded to be analogous to lipopolysaccharide (LPS) [6]. LTA can trigger macrophages to produce huge quantities of inflammatory factors that reveal systemic effects in the general circulation [7]. Apart from the secretion of proinflammatory cytokines TNF, IL-1β, and IL-6, LTA augmented the production of NO [8] and cyclooxygenase (COX)-2; all of these molecules have been found to be induced through phosphorylation of nuclear factor-κB (NF-κB) and mitogen-activated protein kinase (MAPK) [9]. Many studies have shown that NF-κB and MAPK signaling pathways are involved in the expression of proinflammatory mediators. Upon stimulation with LTA or LPS, phosphorylated IκB-α can induce the translocation of NF-κB to the nucleus, where it binds to target genes that encode proinflammatory cytokines [10]. The activation of MAPKs including p38, extracellular signal-related kinase (ERK) 1/2, and c-Jun N-terminal kinase (JNK), upregulates the expression of inflammatory genes [11].

Studies have shown that release of these mediators was implicated in the activation of the NF-κB signaling pathway. Moreover, inflammatory mediator production was also linked with stimulation of the MAPK pathway [12]. Due to the fact that the MAPK and NF-κB signaling pathways are markedly regulated by the expression of pro-inflammatory mediators at the transcriptional level [13], NF-κB and MAPK inhibition has been identified as a potential therapeutic method for treating inflammatory diseases [14]. Activation of reactive oxygen species (ROS) production during inflammation damages major cellular components via inducing oxidative stress [3]. Therefore, oxidative stress and inflammation are closely associated, and they drive the central pathophysiological processes of several diseases [15]. Former studies established that Nrf2 found in the antioxidant pathway plays an important role in oxidative stress-mediated inflammatory disease [16]. Therefore, it is of great interest to advance novel anti-inflammatory bioactive compounds through the upregulation and/or suppression of the Nrf2 and NF-κB pathways, respectively.

Traditional Chinese medicine (TCM) has long been used for the treatment of several human diseases. *Cortex Fraxini* is one of the generally used TCMs. Among the numerous active components found in *C. Fraxini,* esculetin, a dihydroxycoumarin, was explored as the main active ingredient. Several biological activities, such as expectorant, antitussive, anti-inflammatory, antioxidant, antibacterial, have been investigated with esculetin [17,18]. Esculetin has strong therapeutic effect on inhibiting cartilage destruction in rheumatoid arthritis and osteoarthritis [19], diminution of the disease progression of psoriatic mouse skin [20], and reduction of LPS-induced acute lung injury [21]. Esculetin has several pharmacological effects, and it has been a promising lead for medicinal chemists developing novel drug candidates; however, the beneficial effects and the underlying mechanism of esculetin on LTA-induced macrophages remain unclear. In this study, we evaluated whether esculetin exhibits anti-inflammatory activity by activating the Nrf2 signaling molecule, which conquers the NF-κB signaling cascades.

## 2. Results

### 2.1. Esculetin Did Not Affect Viability in RAW Cells

**Figure 1:**
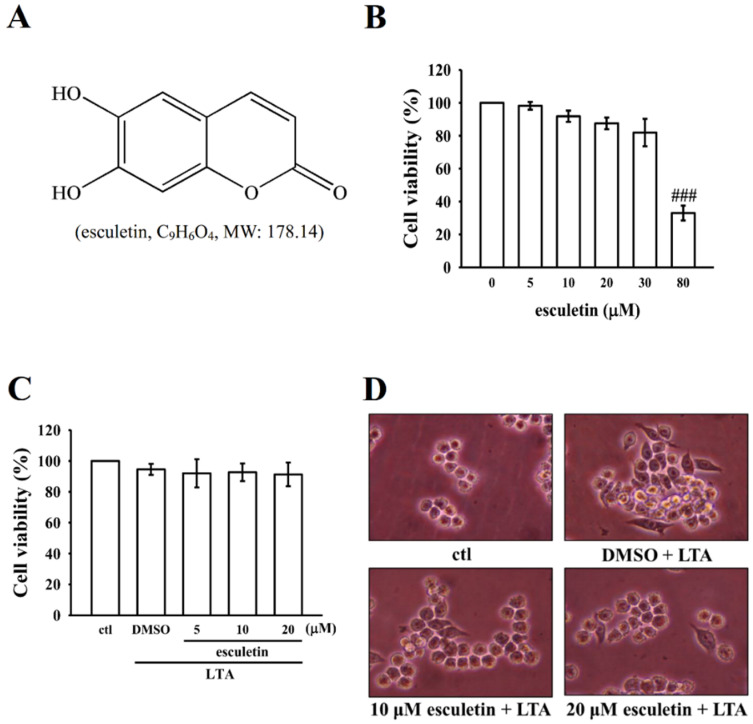
(**A**) The chemical structure of esculetin. 3-(4,5-dimethylthiazol-2-yl)-2,5-diphenyltetrazolium bromide (MTT) cell viability assay of (**B**) esculetin (5–80 µM) alone and (**C**) esculetin pretreated lipoteichoic acid (LTA)-induced RAW 264.7 cells, and (**D**) morphology of esculetin (10 and 20 μM) in LTA-induced RAW cells as described in the Methods section. Data are presented as the means ± standard error (SEM, *n* = 4); ^###^ *p* < 0.001 compared with the control group.

To assess the anti-inflammatory potential of esculetin, NO production and iNOS expression were measured in cultured RAW 264.7 cells treated with LTA to induce inflammation, then consequently treated with esculetin. As shown in Figure 1A–C, esculetin alone or with LTA did not induce cytotoxicity in RAW cells. However, esculetin induced a significant reduction in cell viability at 80 µM concentration. Moreover, as clearly shown by the cell morphology images in Figure 1D, the ideal concentrations of 10 and 20 µM esculetin did affect cell morphology; hence, these concentrations were chosen for further study.

### 2.2. Esculetin Inhibits LTA-Induced NO Production and iNOS Expression

**Figure 2:**
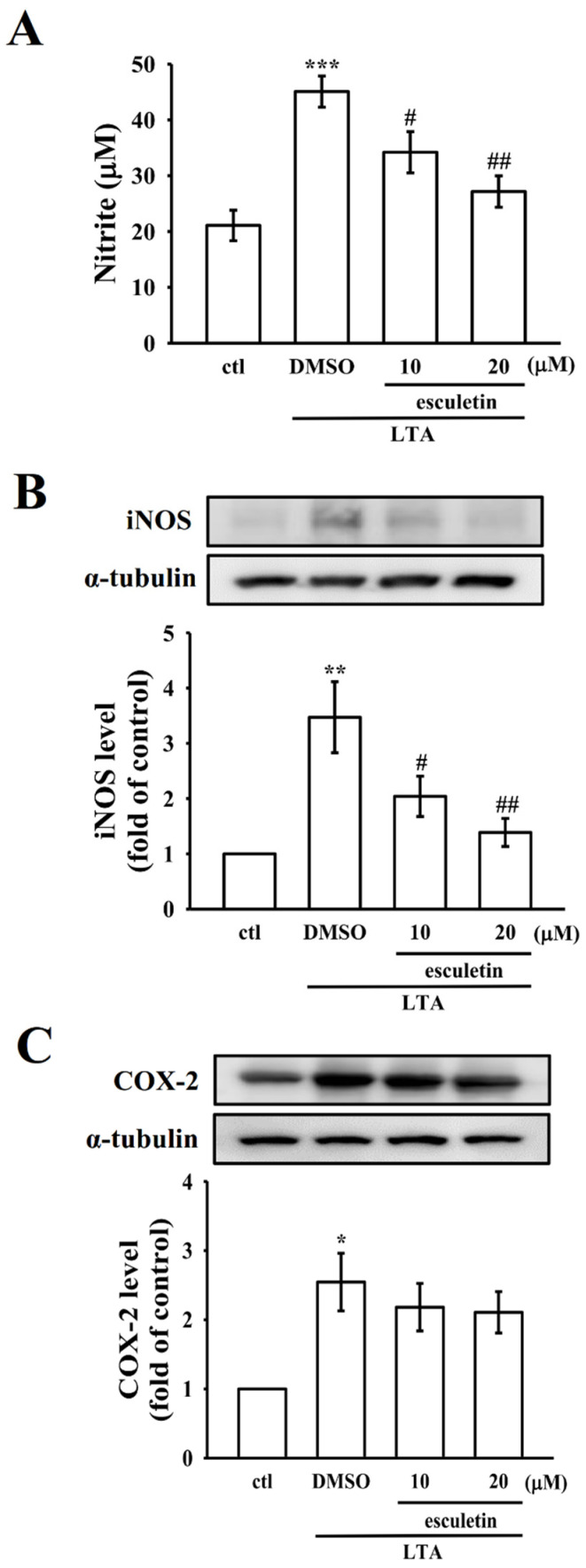
Effects of esculetin on nitric oxide (NO) production, and on the expression of inducible nitric oxide synthase (iNOS), and cyclooxygenase-2 (COX-2) in LTA-stimulated RAW cells. Cells were pretreated with esculetin (10 and 20 µM) for 1 h and then stimulated by LTA (10 µg/mL) for 24 h. (**A**) NO was measured using Griess reagent. (**B**,**C**) The levels of (**B**) iNOS and (**C**) COX-2 protein expression were evaluated as described in the Methods section. Data are presented as the means ± SEM (*n* = 4); * *p* < 0.05, ** *p* < 0.01, and *** *p* < 0.001, compared with the control group; ^#^ *p* < 0.05 and ^##^ *p* < 0.01, compared with the LTA group.

Figure 2A shows that an intense elevation of NO, a pro-inflammatory mediator that can induce systemic inflammatory events [22], led to production in LTA-exposed cells being significantly and concentration-dependently suppressed by esculetin treatment. NO synthesis is proposed to be regulated by a rate-limiting enzyme, inducible nitric oxide synthase (iNOS) [22]. Therefore, the expression of iNOS was detected to examine if esculetin inhibits NO production via the modulation of iNOS expression. As shown in Figure 2B, esculetin inhibits LTA-induced protein expression of iNOS in RAW264.7 cells, inferring that esculetin inhibited LTA-induced NO production by inhibiting the activity of iNOS. COX-2, an inducible enzyme, enhances the reaction for the transformation of arachidonic acid (AA) to prostaglandin H2 (PGH2), which is a precursor of several inflammatory mediators, such as PGE2 and prostacyclin, during inflammation. However, esculetin (10 and 20 uM) had no effect on the basal COX activity in LTA-stimulated RAW cells (Figure 2C).

### 2.3. Esculetin Did Not Affect MAPK Pathways to Exert Its Anti-Inflammatory Effects in LTA-Induced RAW 264.7 Cells

**Figure 3:**
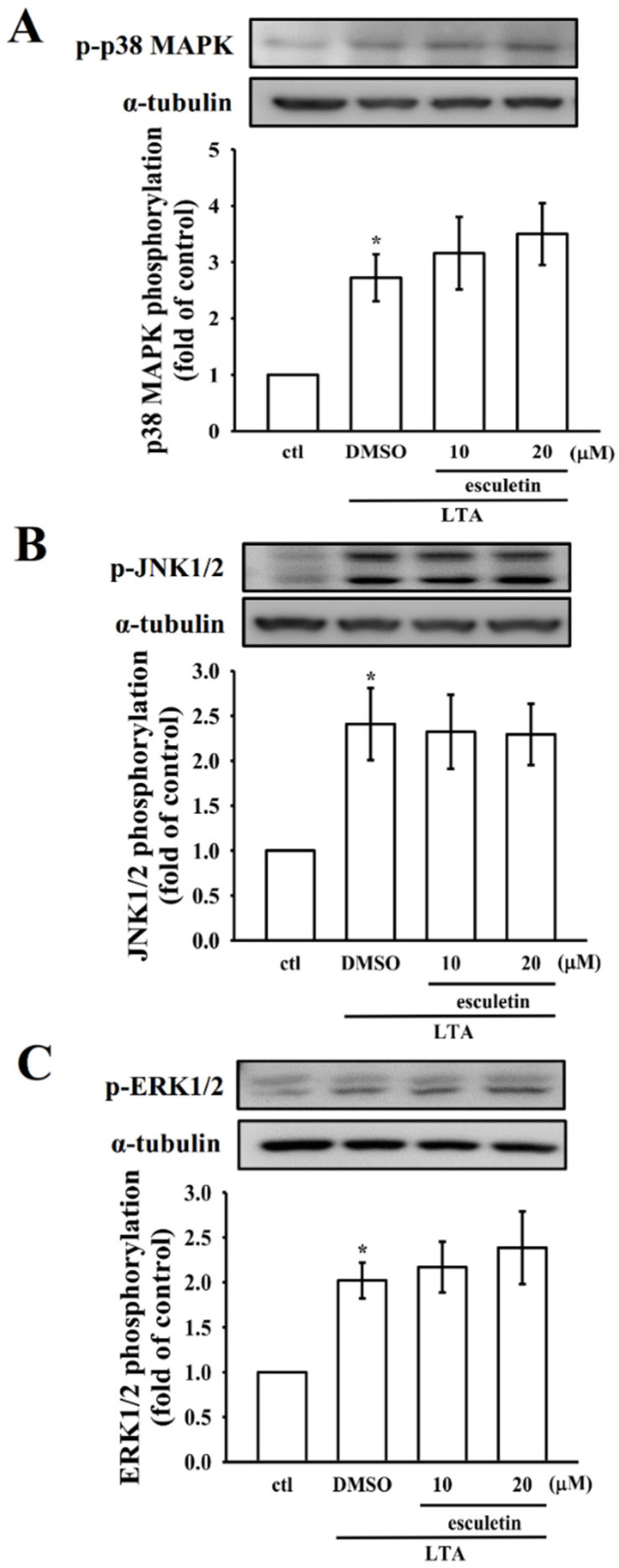
Influence of esculetin on LTA-induced phosphorylation of p38 mitogen-activated protein kinase (p38 MAPK), c-Jun NH2-terminal kinase (JNK), and extracellular signal-regulated kinase (ERK) in RAW cells. (**A–C**) Cells were treated with 0.1% Dimethyl sulfoxide (DMSO) or esculetin (10 and 20 μM) for 1 h, followed by LTA (10 μg/mL) for 1 h, and the phosphorylation of (**A**) p38 MAPK, (**B**) JNK1/2, and (**C**) ERK1/2 were evaluated by immunoblotting assay as described in the Methods section. Data are presented as the means ± SEM (*n* = 4). * *p* < 0.05, compared with the control group.

Studies have been shown that inflammatory mediator expression in LTA-treated macrophages is controlled by MAP kinases. This study examined esculetin’s effect on MAPK signaling. As shown in Figure 3, RAW cells stimulated with LTA significantly promoted the expression of phosphorylated ERK1/2, JNK1/2 and p38 MAPK compared with the control cells. Additionally, esculetin pretreatment did not induce any significant changes in LTA-induced MAPK phosphorylation (Figure 3A–C), indicating that esculetin shows its anti-inflammatory effect through MAPK-independent mechanisms.

### 2.4. Esculetin Regulates NF-κB Pathway to Exert its Anti-Inflammatory Effects

**Figure 4:**
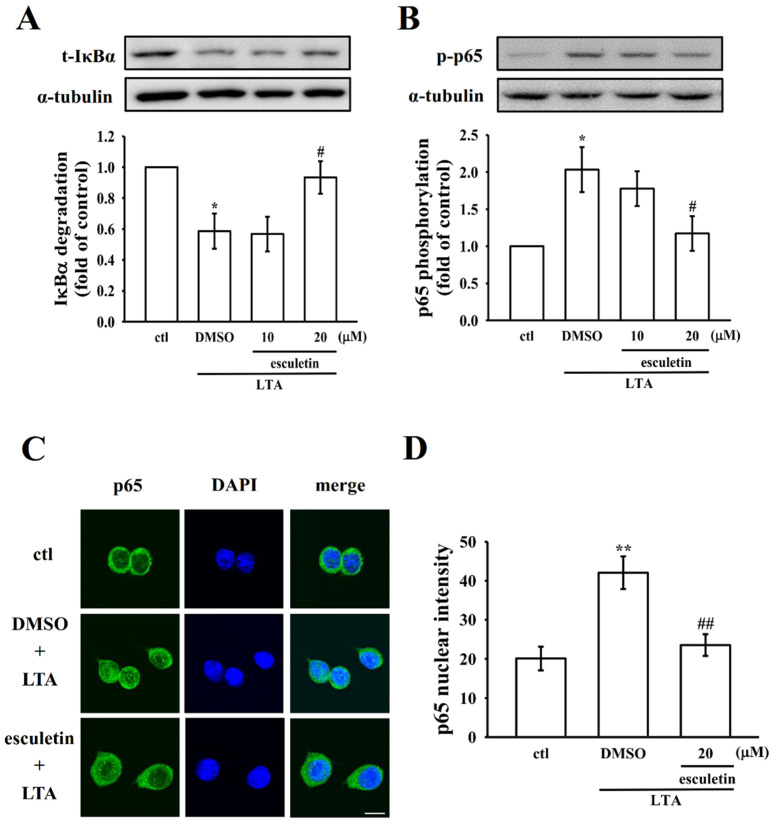
Esculetin regulates the nuclear factor kappa B (NF-κB) signaling pathway that was induced by LTA in RAW cells. Cells were treated with esculetin (10 and 20 µM) and LTA (10 µg/mL) for 1 h. Western blotting assay was performed to detect (**A**) the degradation of IκBα and (**B**) the phosphorylation of p65 in LTA-induced RAW cells. (**C**) Esculetin inhibited LTA-induced p65 nuclear translocation (scale bar = 10 μm). (**D**) Data were graphed by pooling multiple images, with each individual data point corresponding to the mean fluorescence intensity of each individual cell nucleus. Data are presented as the means ± SEM (*n* = 4). * *p* < 0.05 and ** *p* < 0.01, compared with the control group; ^#^ *p* < 0.05 and ^##^ *p* < 0.01, compared with the LTA group.

The NF-κB pathway is reported to regulate the production of pro-inflammatory cytokines and mediators, which are key drivers of the inflammatory response [23]. Consequently, in order to observe the effect of esculetin in inhibition of NF-κB signaling, as shown in Figure 4A,B, esculetin in LTA-induced RAW cells inhibited the degradation and phosphorylation, respectively, of IκBα and p65. As shown in Figure 4C,D, it was established that esculetin inhibited p65 translocation from cytosol into nucleus. These results suggest that esculetin has an anti-inflammatory effect by inhibiting the NF-κB pathway.

### 2.5. Effects of Esculetin on Nrf2 in LTA-Induced RAW Cells

**Figure 5:**
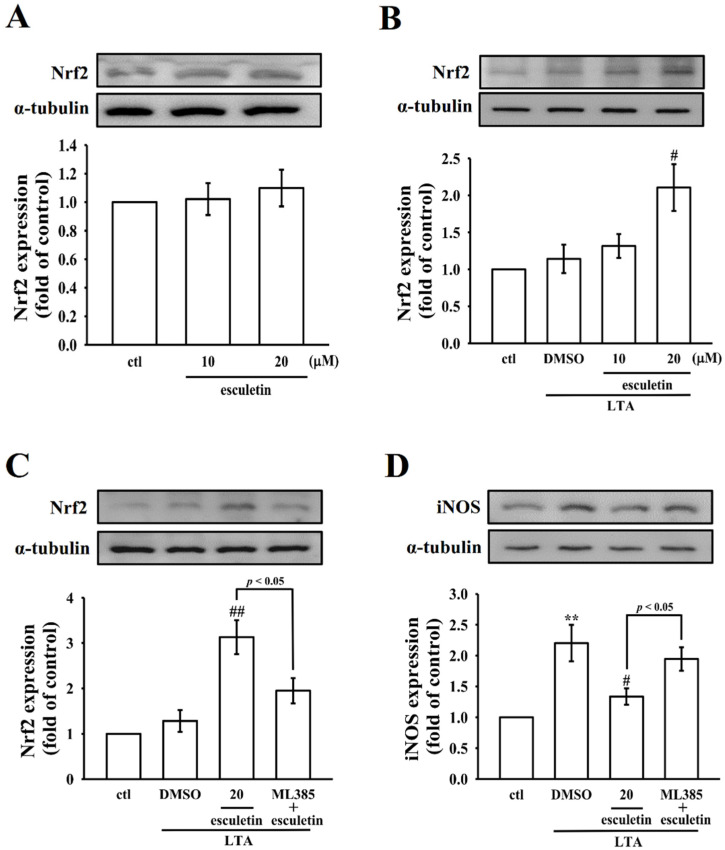
Esculetin enhances antioxidant defense molecules Nrf2 in LTA-induced RAW cells. Cells were treated with (**A**) 0.1% DMSO or esculetin (10 and 20 µM) alone, and (**B**) treated with 0.1% DMSO or esculetin (10 and 20 µM) for 1 h followed by LTA (10 µg/mL) for 24 h. (**C**,**D**) Cells were treated with 0.1% DMSO, 20 uM esculetin, or 10 uM ML385 for 1 h, followed by LTA for 24 h, and (**C**) Nrf2 and (**D**) iNOS expression were determined by immunoblotting assay, as described in the Methods. Data are presented as the means ± SEM (*n* = 4). ** *p* < 0.01, compared with the control group; ^#^ *p* < 0.05 and ^##^ *p* < 0.01, compared with the LTA group.

In normal states, cytoplasmic Nrf2 is in inactivated form upon binding to its inhibitor, Keap1. Upon activation, Nrf2 translocates to the nucleus after detaching from Keap1 and binds directly to antioxidant responsive elements (AREs) in target promoters, promoting the expression of several antioxidant genes [24]. Studies have validated the relationship between Nrf2 and NF-κB signaling [25]. Since our results demonstrated that esculetin inhibits NF-κB activation, this study further sought to determine if esculetin also influences Nrf2 signaling. We found that pretreatment with esculetin alone did not affect Nrf2 expression, as shown in Figure 5A. However, esculetin pretreatment in LTA-induced cells significantly elevated the expression of Nrf2 (Figure 5B). To further verify if esculetin activates Nrf2 in raw 264.7 macrophage cells to show its anti-inflammatory responses, the inhibitory effect of ML385, an inhibitor of Nrf2 was evaluated. As shown in Figure 5C,D, ML385 reversed the esculetin-regulated Nrf2 and iNOS expression in LTA-induced RAW cells. These findings suggest that esculetin activates the Nrf2 signaling molecule in addition to its suppressing effect on the NF-κB pathway.

### 2.6. Scavenging Activity of the 1,1-Diphenyl-2-Picrylhydazyl (DPPH) Radical

**Figure 6:**
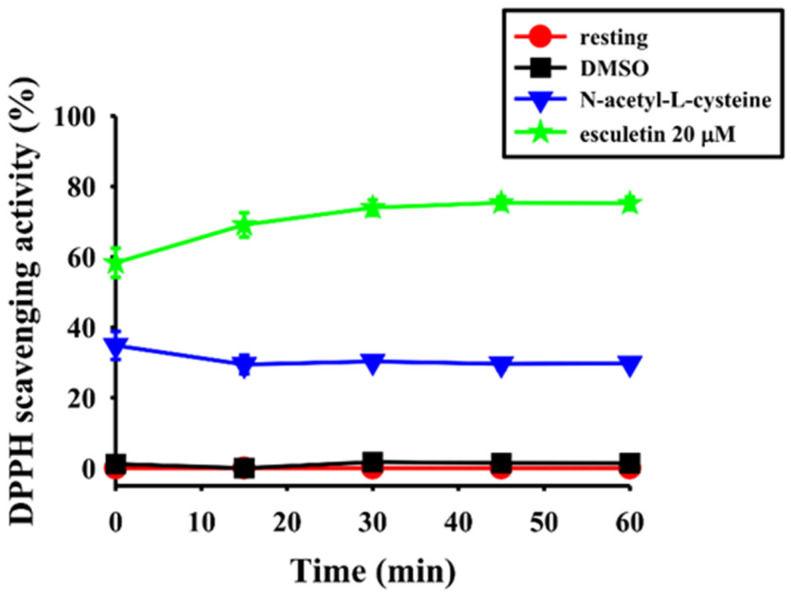
Esculetin scavenges 2,2-diphenyl-1-picrylhydrazyl (DPPH) radical formation in RAW cells. DPPH–methanol (300 µM) was mixed with esculetin (20 µM) and then incubated for 30 min. The absorbance at 517 nm was determined using a spectrophotometer.

DPPH radical-scavenging activity of esculetin was measured to check whether esculetin has anti-oxidative activities in association with its anti-inflammatory effects. Esculetin exhibited potent free radical-scavenging activities compared with the positive control, N-acetylcysteine (Figure 6). DPPH radical scavenging rates of NAC rose gradually with time, whereas at 10-60 min time intervals, DPPH radical scavenging rates of esculetin were increased progressively. Therefore, esculetin could be considered as an exceptional DPPH radical scavenger. The mechanism of the higher scavenging activity of esculetin is due to an electron-donating ability from the two attached phenolic hydroxyl groups, as explained by Chen et al. [26].

## 3. Discussion

**Figure 7:**
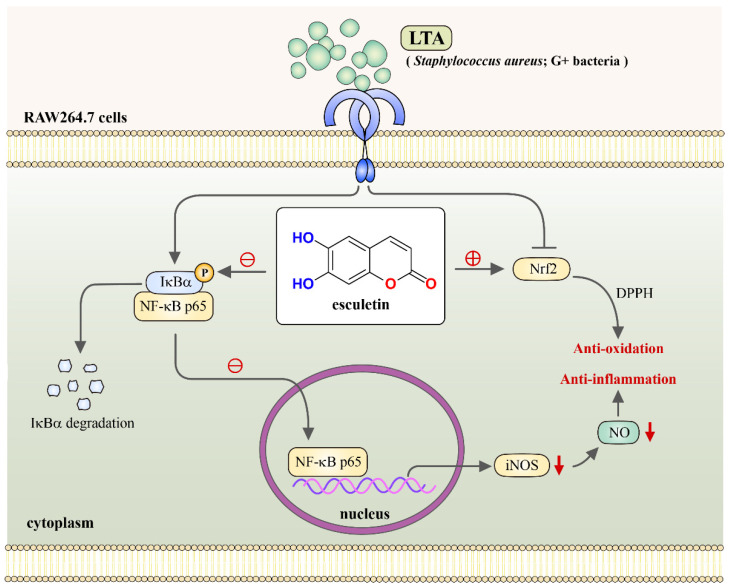
Schematic of the anti-inflammatory and anti-oxidative activities of esculetin in vitro. Esculetin exerts anti-inflammatory activity by inhibiting the production of NO via suppression of NF-κB, and enhancing Nrf2 expression. LTA, lipoteichoic acid; NO, nitric oxide; iNOS, inducible nitric oxide synthase; NF-κB, nuclear factor kappa B; Nrf2, nuclear factor erythroid 2-related factor.

LTA is a major endotoxin that plays an important role in infection and post-infection pathological progressions produced by Gram-positive bacteria [27]. This study established that LTA induces the expression of proinflammatory mediators in RAW macrophages. Moreover, LTA was able to intensify the transcriptional activity of NF-κB, as evidenced by an increase in the respective degradation and phosphorylation of IκBα and NF-κB p65. Plants and herbal based natural drugs have fascinated researchers and biomedical industries due to their extensive immunomodulatory activities against endotoxin-induced inflammations. In our previous study, we established that esculetin, a bioactive 6,7-dihydroxy derivative of coumarin, possesses antiplatelet activity via hampering the PLCγ2-PKC cascade, hydroxyl radical formation, and Akt activation; thus, we suggested that esculetin may act as a crucial therapeutic agent for preventing thromboembolic diseases. In this study, the anti-inflammatory mechanism of esculetin was investigated in RAW cells. Precisely, we studied the impact of esculetin on Nrf2 and NF-κB signaling. We found that esculetin exhibits a potent inhibitory effect on NO, iNOS and DPPH radicals via modulating Nrf2 and NF-κB in LTA-induced RAW cells without affecting cell viability (Figure 7). These results demonstrated that extreme inflammation destruction by esculetin is complicated and happens, at least in part, via Nrf2-mediated inhibition of NF-κB signaling.

Stimulated macrophages fulfill a vital role in the inflammatory processes [28] and cause rapid activation of inflammatory stimulators. For instance, activation of NO and PGE2 and proinflammatory cytokines TNF-α, IL-1β, and IL-6 results in the advancement of several inflammatory disorders [25]. Cheng et al. [29] showed recovered lung injury in esculetin-treated animals with restrained production of IL-1β, IL-6, TNF-α, and iNOS in LPS-induced sepsis models [30]. Studies have reported that some small molecular weight plants and microbial peptides presented anti-inflammatory effects by affecting the production of PGE2 and down-regulating genes associated with inflammation in RAW 264.7 cells [30]. This work discovered that esculetin remarkably impeded the production of NO and iNOS, but it did not respond to COX-2 expression. Consistent with this data, a recent study found that YD1, a novel antimicrobial peptide isolated from kimchi, extraordinarily hindered the production of NO by inhibiting the expression of iNOS [31]. Isolated benzoic and cinnamic acid derivatives from sorghum grains have been reported to inhibit the production of NO in LPS-induced RAW 264.7 macrophages with the associated reduction of iNOS and COX-2 expressions [32].

iNOS is a target gene of NF-κB, and NF-κB plays a pivotal role in the development of inflammation by activating the transcription of inflammatory genes. COX-2 expression is regulated at both the transcriptional and post-transcriptional levels. The transcriptional activation of COX-2 is mediated by the binding of the inducible transcription factors. The specific factors involved in COX-2 activation depend on both the cell type and the stimulus. Thus, NF-κB contributes to COX-2 induction in some, but not all, cell types. In addition, Lee et al. [33] also used plant extraction to investigate their anti-inflammation and antioxidant effects, and their data is consistent with our data. These data confirm the claim of Wadleigh et al. [34] that endotoxin engages NF-κB, and the contribution of NF-κB to COX-2 transactivation in mouse macrophages stimulated with endotoxin may be dispensable. Moreover, NF-κB activation also plays a key role in the production of inflammatory cytokines [35]. It has been reported that the inhibition of NF-κB activation can decrease its downstream inflammatory mediator [36]. In normal physiological conditions, NF-κB is impounded in the cytoplasm by its inhibitor IκB, and a study has established that NF-κB is a typical transcription factor in response to LTA activation [7]. LTA binds with TLR2 and activates NF-κB by protein kinases and is then translocated to nuclei from the cytoplasm [37]. NF-κB normalizes certain gene expressions that regulate cell proliferation, differentiation, and death [38]. One of our previous studies showed that LTA induced COX-2 expression in epithelial cells via IκB degradation and successive p65 NF-κB translocation [39]. As the expression of pro-inflammatory mediators is strongly controlled at a transcriptional level via the MAPK and NF-κB signaling pathways [40], the targeted inhibition of the activity of NF-κB and MAPK has been recognized as a potential therapeutic method for inflammatory diseases [14]. In this study, we demonstrated that LTA stimulation expressively increased the degradation of IκBα and p65 phosphorylation. Moreover, esculetin treatment reserved IκBα degradation and p65 phosphorylation. Nevertheless, MAPK, including ERK, JNK and p38 MAPK, have been demonstrated to play a crucial role in signal transduction pathways, regulation of cytokine release, and participation in the activation of NF-κB in LPS-induced inflammation [41]. Surprisingly, in this study, esculetin pretreatment did not alter all of those MAPK signaling molecules. These results indicated that NF-κB signaling pathways, but not MAPK, could be the chief causes of esculetin diminution of LTA-induced inflammation.

It is anticipated that Nrf2 and NF-κB signaling pathways conjoin to conserve the physiological homeostasis of cellular redox status and to control the cellular response during stress and inflammatory situations. Based on the functional viewpoint, Nrf2 adversely regulates the NF-κB signaling pathway via multiple mechanisms. Primarily, Nrf2 hinders oxidative stress-mediated NF-κB activation by decreasing the intracellular ROS levels [42]. Moreover, Nrf2 prevents IκBα degradation and inhibits NF-κB nuclear translocation [43]. Hong et al. [44] have reported that esculetin attenuated LPS-induced production of pro-inflammatory mediators in RAW264.7 cells by suppressing the activation of NF-κB signaling and the generation of ROS. Consistently, as this study found that esculetin significantly inhibited p65 phosphorylation and translocation, we suspected that this compound may enhance Nrf2 expression in LTA-stimulated RAW cells. As expected, esculetin in LTA-exposed RAW cells significantly enhanced the expression of Nrf2. In addition, esculetin could reduce the ROS production induced by LTA by about 21% and 59% (10 µM and 20 µM, n=3, data not shown). It was clearly indicated that esculetin’s preventive effect against LTA-induced IκBα degradation and nuclear translocation of NF-κB may partially be due to its enhancing effects on Nrf2 expression, as explored by Yerra et al. [43]. Another relevant study discovered that an antimicrobial peptide, YD1, activates Nrf2 signaling, which inhibits the phosphorylation of IκBα and, following NF-κB activation, subsequently suppresses inflammation [32]. To investigate whether the treatment of esculetin depends on Nrf2, the inhibition of Nrf2-ML385 was used in cells. The results showed that ML385 increased iNOS protein expression, and the data are consistent with Yao et al. [45]. This outcome further confirmed the contribution of Nrf2-mediated NF-κB inhibition to the anti-inflammatory effects of esculetin.

Free radicals such as superoxide radical (O2-), hydroxyl radical (OH-), peroxyl radical (ROO) and nitric oxide radical (NO) are reported to widely contribute in biological metabolism [46]. Conversely, oxidative stress, described as an imbalance between the over-production of free radicals and antioxidants, is the primary cause of numerous chronic diseases [47]. In the present study, the capability to inhibit DPPH production by esculetin seemed to be expressively more potent than a standard antioxidant, N-acetyl-L-cysteine (NAC). This result is more consistent with an earlier study that found the extracts of sorghum quenched by > 90% of DPPH radicals than of other radicals [48]. Our result is also equivalent to another study, where it was reported that three different genotype sorghum flours quenched 90% of DPPH [49]. The effect of antioxidants on DPPH was believed to be due to their hydrogen donating ability; the more hydroxyl substitution, the stronger the antioxidant activities. Considering its molecular structure, we estimate that the potent scavenging activities of esculetin may principally be due to the hydrogen donating ability of phenolic hydroxy groups in the esculetin molecule that can convert the DPPH radical into constant molecules effectively.

## 4. Materials and Methods

### 4.1. Materials

Esculetin (≥98%) and ML385 were obtained from Cayman (Ann Arbor, MI, USA). Dimethyl sulfoxide (DMSO), lipoteichoic acid (LTA), N-acetyl L-cysteine (NAC) and 2,2-diphenyl-1-picrylhydrazyl (DPPH) were obtained from Sigma (St. Louis, MO, USA). Anti-iNOS (sc-650) and polyclonal antibody (pAb) were purchased from Santa Cruz Biotechnology (Dallas, TX, USA). The anti-phospho-c-JNK (Thr183/Tyr185, 9251), anti-phospho-p44/p42 ERK (Thr202/Tyr204, 9101) and anti-phospho-p38 MAPK (Thr180/Tyr182, 9211) pAbs, and anti-phospho-p65 (Ser536, 3033), anti-p65 (4764) and anti-IκBα (4812) mAbs were purchased from Cell Signaling (Danvers, MA, USA). Anti-Nrf2 (GTX103322) pAbs was purchased from GeneTex (Irvine, CA, USA). Fetal bovine serum (FBS), Dulbecco’s modified Eagle medium (DMEM), L-glutamine penicillin/streptomycin, and anti-α-tubulin monoclonal antibodies (mAbs) were purchased from Invitrogen (Thermo Fisher Scientific, Waltham, MA, USA). Amersham (Buckinghamshire, UK) supplied horseradish peroxidase (HRP)-conjugated donkey anti-rabbit immunoglobulin G (IgG) and sheep anti-mouse IgG.

### 4.2. Cell Culture and Viability Assay

RAW 264.7 cells were purchased from ATCC (ATCC number: TIB-71). Cells were cultured in DMEM medium added with 10% FBS, 100 U/mL penicillin G and 100 mg/mL streptomycin at 37 °C in a humidified atmosphere of 5% CO_2_/95% air.

Cells at a density of 1 × 10^5^ cells per well were incubated and then treated with different concentrations of esculetin (5–80 μM) or solvent control (0.1% DMSO) for 1 h and then stimulated with LTA (10 μg/mL) for 24 h. The cytotoxicity of esculetin was measured by using MTT assay. The specified formula of absorbance of treated-cells/absorbance of control cells × 100% was applied to calculate the cell viability index. The absorbance of each well was read at 570 nm using an MRX absorbance reader (Dynex Technologies, Chantilly, VA, USA).

### 4.3. Measurement of Nitric Oxide Concentration

The production of NO was measured by Griess method with microplate. To this, cells were treated with esculetin (10 and 20 μM) or 0.1% DMSO for 1 h and then stimulated with LTA (10 μg/mL) for 24 h. The conditioned supernatants were collected and mixed with an equal volume of Griess reagent (1% sulphanilamide and 0.1% naphthalenediamine dissolved in 2.5% phosphoric acid). The absorbance of each sample was read at 540 nm by an MRX absorbance reader. Sodium nitrite was used as a standard.

### 4.4. Western Blotting

RAW 264.7 cells were harvested and lysed by using an ice-cold lysis buffer. The estimated total protein of 50 μg in each sample was electrophoretically separated by 12% sodium dodecyl sulphate-polyacrylamide gel electrophoresis (SDS-PAGE) and transferred onto PVDF membranes (0.45 μm). The membranes were blocked with 5% skimmed milk in TBST buffer (10 mM Tris-base, 100 mM NaCl and 0.01% Tween 20) for 30 min and then incubated with various primary antibodies of target proteins for 2 h. After the incubation, membranes were subjected to HRP-conjugated donkey anti-rabbit IgG or sheep anti-mouse IgG at room temperature for 1 h. The density of protein bands was obtained by the Biolight Windows Application, V2000.01 (Bio-Profil, Vilber Lourmat, France).

### 4.5. Immunofluorescence Staining Assay

In 6-well plates, cells at a density of 5 × 10^4^ per well were cultured on cover slips and treated with 0.1% DMSO or 20 μM esculetin with or without LTA stimulation for 1 h. The cover slips were washed with PBS and then fixed with 4% paraformaldehyde at room temperature for 10 min. Cells were permeabilized with 0.1% Triton X-100 for 10 min and blocked with 5% BSA for 30 min. After the incubation, cells were titrated with the respective primary antibody overnight at 4 °C, subsequently washed with PBS three times, and titrated with secondary antibody at room temperature for 1 h. The samples were stained with 4,6-diamidino-2-phenylindole (DAPI, 30 μM) and mounted using a mounting buffer (Vector Laboratories) on a glass slice. The observations were spotted under a Leica TCS SP5 confocal spectral microscope imaging system using an argon or krypton laser (Mannheim, Germany).

### 4.6. Determination of DPPH Free Radical Scavenging Activity

According to the method described by Sharma and Bhat [50], the scavenging activity of DPPH radical was tested with minor modifications. Briefly, DPPH–methanol (300 µM) was mixed with esculetin (20 µM) or 0.1% DMSO and then incubated for 30 min. The absorbance was read at 517 nm using a spectrophotometer (UV-160; Shimadzu, Kyoto, Japan). NAC was used as a positive control. DPPH scavenging activity (%) was calculated as (A2 − A1)/A2 × 100%, where A1 is the absorbance of esculetin, and A2 is the absorbance of DPPH.

### 4.7. Statistical Analysis

Results are shown as the means ± standard error (SEM). Each experiment was repeated at least four times. Statistical analysis was performed using one-way analysis of variance (one-way ANOVA). Significant differences among the groups were compared using the Newman–Keuls method [51]. The *p*-values < 0.05 were considered statistically significant.

## 5. Conclusions

This study established that esculetin possesses potent anti-inflammatory activity in LTA-stimulated RAW cells via triggering on Nrf2-mediated inhibition of NF-κB signaling pathways with independent mechanism of MAPK signaling. This inhibitory effect of esculetin resulted in the solid mitigation of proinflammatory mediators iNOS, NO and DPPH radical quenching. Altogether, this work can recommend esculetin as a conceivable novel anti-inflammatory agent, and this study could help define if esculetin could be an ideal therapeutic for inhibiting and treating inflammation-related disorders and oxidative stress.

## Data Availability

All data generated or analyzed during this study are included in this published article.

## Sample Availability

Samples of the compounds are not available from the authors.
